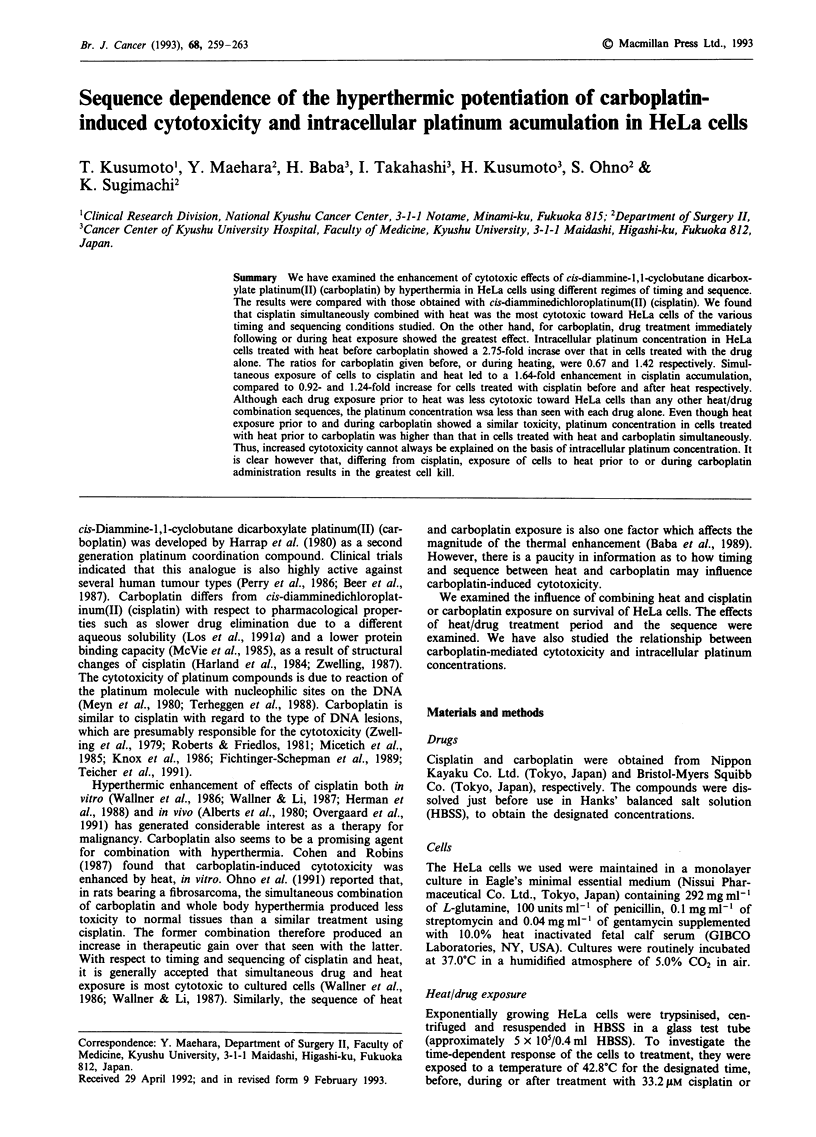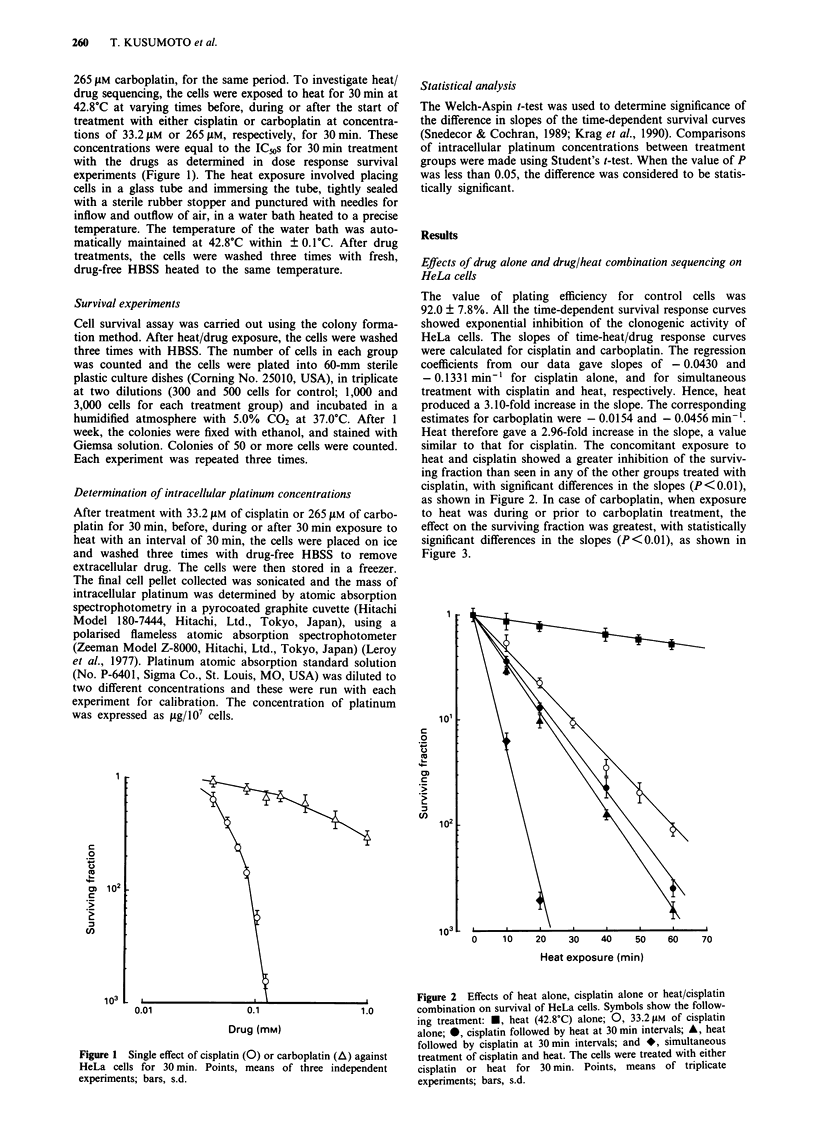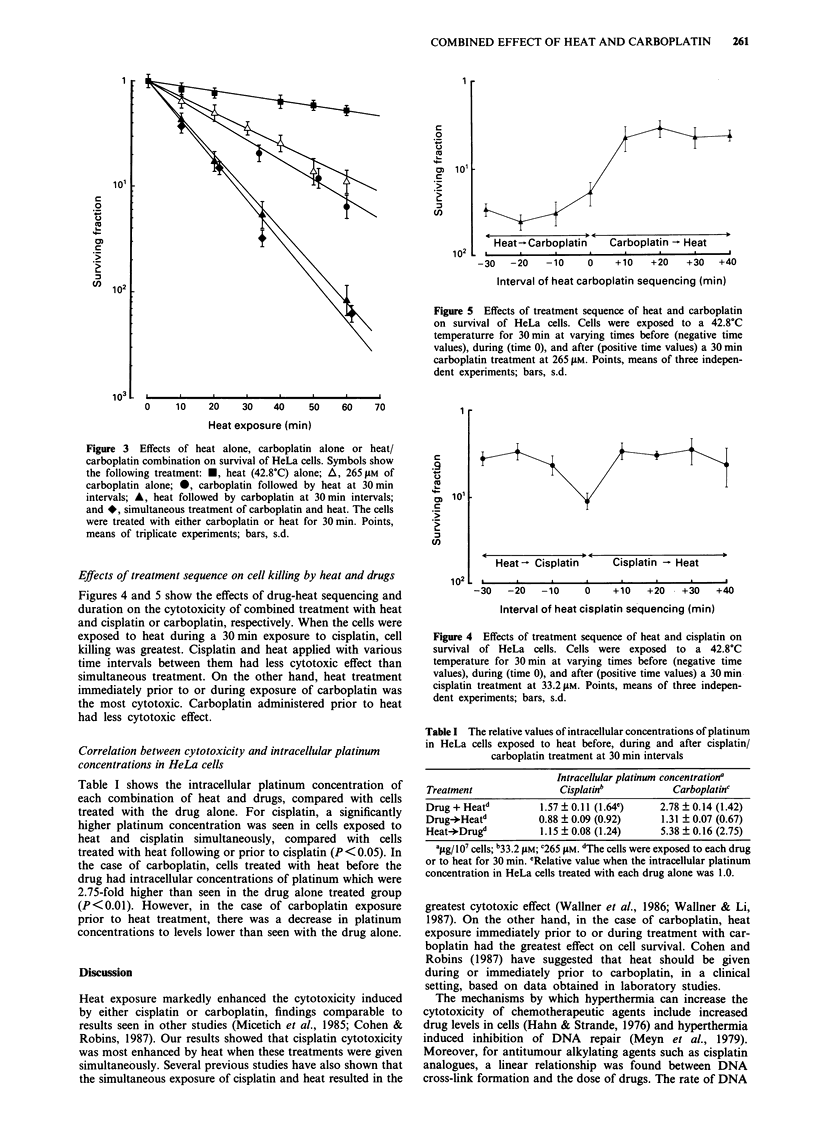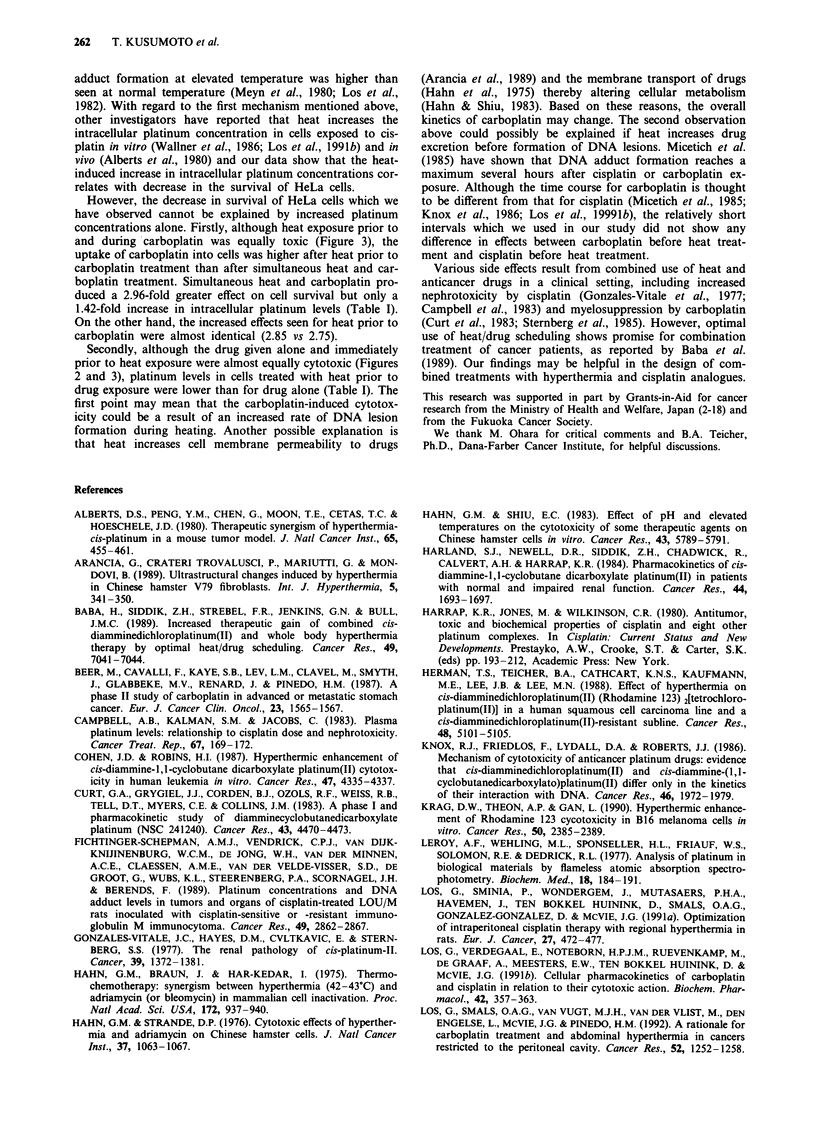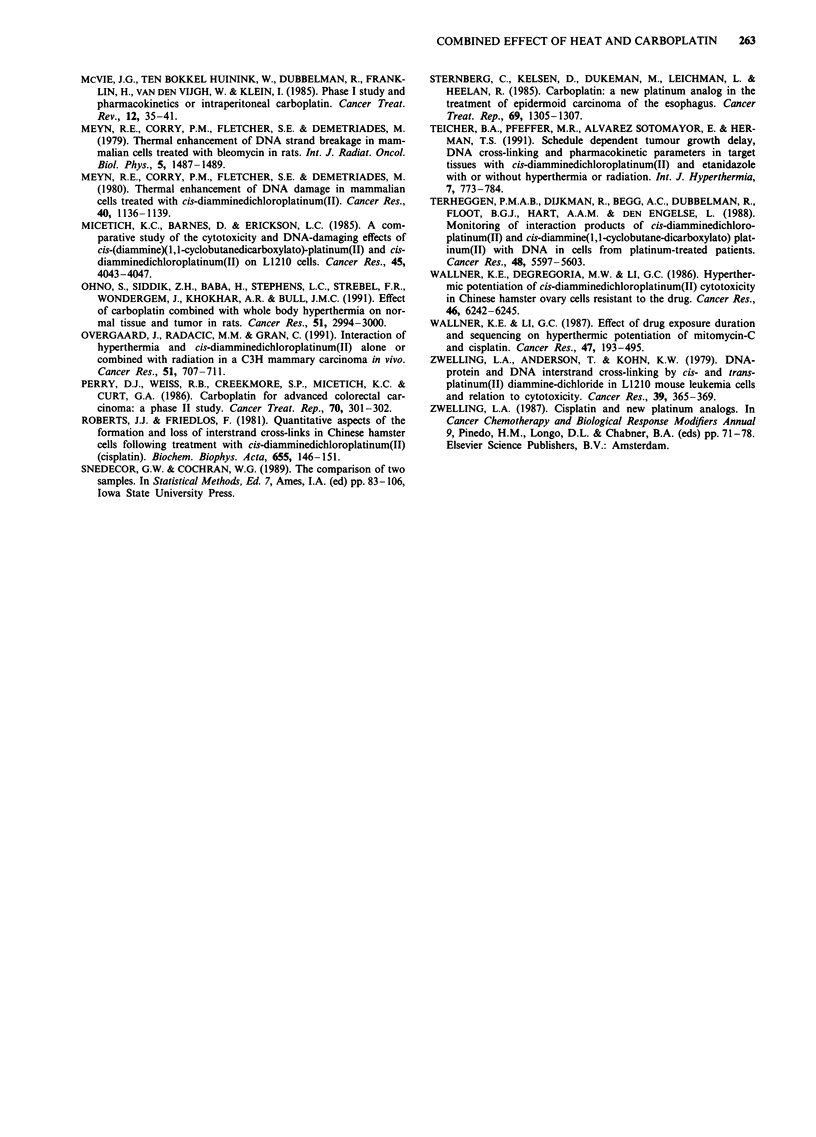# Sequence dependence of the hyperthermic potentiation of carboplatin-induced cytotoxicity and intracellular platinum accumulation in HeLa cells.

**DOI:** 10.1038/bjc.1993.324

**Published:** 1993-08

**Authors:** T. Kusumoto, Y. Maehara, H. Baba, I. Takahashi, H. Kusumoto, S. Ohno, K. Sugimachi

**Affiliations:** Clinical Research Division, National Kyushu Cancer Center, Fukuoka, Japan.

## Abstract

We have examined the enhancement of cytotoxic effects of cis-diammine-1,1-cyclobutane dicarboxylate platinum(II) (carboplatin) by hyperthermia in HeLa cells using different regimes of timing and sequence. The results were compared with those obtained with cis-diamminedichloroplatinum(II) (cisplatin). We found that cisplatin simultaneously combined with heat was the most cytotoxic toward HeLa cells of the various timing and sequencing conditions studied. On the other hand, for carboplatin, drug treatment immediately following or during heat exposure showed the greatest effect. Intracellular platinum concentration in HeLa cells treated with heat before carboplatin showed a 2.75-fold increase over that in cells treated with the drug alone. The ratios for carboplatin given before, or during heating, were 0.67 and 1.42 respectively. Simultaneous exposure of cells to cisplatin and heat led to a 1.64-fold enhancement in cisplatin accumulation, compared to 0.92- and 1.24-fold increase for cells treated with cisplatin before and after heat respectively. Although each drug exposure prior to heat was less cytotoxic toward HeLa cells than any other heat/drug combination sequences, the platinum concentration was less than seen with each drug alone. Even though heat exposure prior to and during carboplatin showed a similar toxicity, platinum concentration in cells treated with heat prior to carboplatin was higher than that in cells treated with heat and carboplatin simultaneously. Thus, increased cytotoxicity cannot always be explained on the basis of intracellular platinum concentration. It is clear however that, differing from cisplatin, exposure of cells to heat prior to or during carboplatin administration results in the greatest cell kill.


*Br. J. Cancer* (1993), **68**, 259–263 © Macmillan Press Ltd., 1993

### Sequence dependence of the hyperthermic potentiation of carboplatin-induced cytotoxicity and intracellular platinum acumulation in HeLa cells

T. Kusumoto[1], Y. Maehara[2], H. Baba[3], I. Takahashi[3], H. Kusumoto[3], S. Ohno[2] & K. Sugimachi[2]

[1]*Clinical Research Division, National Kyushu Cancer Center, 3-1-1 Notame, Minami-ku, Fukuoka 815;* [2]*Department of Surgery II,* [3]*Cancer Center of Kyushu University Hospital, Faculty of Medicine, Kyushu University, 3-1-1 Maidashi, Higashi-ku, Fukuoka 812, Japan.*

**Summary** We have examined the enhancement of cytotoxic effects of *cis*-diammine-1,1-cyclobutane dicarboxylate platinum(II) (carboplatin) by hyperthermia in HeLa cells using different regimes of timing and sequence. The results were compared with those obtained with *cis*-diamminedichloroplatinum(II) (cisplatin). We found that cisplatin simultaneously combined with heat was the most cytotoxic toward HeLa cells of the various timing and sequencing conditions studied. On the other hand, for carboplatin, drug treatment immediately following or during heat exposure showed the greatest effect. Intracellular platinum concentration in HeLa cells treated with heat before carboplatin showed a 2.75-fold incrase over that in cells treated with the drug alone. The ratios for carboplatin given before, or during heating, were 0.67 and 1.42 respectively. Simultaneous exposure of cells to cisplatin and heat led to a 1.64-fold enhancement in cisplatin accumulation, compared to 0.92- and 1.24-fold increase for cells treated with cisplatin before and after heat respectively. Although each drug exposure prior to heat was less cytotoxic toward HeLa cells than any other heat/drug combination sequences, the platinum concentration wsa less than seen with each drug alone. Even though heat exposure prior to and during carboplatin showed a similar toxicity, platinum concentration in cells treated with heat prior to carboplatin was higher than that in cells treated with heat and carboplatin simultaneously. Thus, increased cytotoxicity cannot always be explained on the basis of intracellular platinum concentration. It is clear however that, differing from cisplatin, exposure of cells to heat prior to or during carboplatin administration results in the greatest cell kill.

*cis*-Diammine-1,1-cyclobutane dicarboxylate platinum(II) (carboplatin) was developed by Harrap *et al.* (1980) as a second generation platinum coordination compound. Clinical trials indicated that this analogue is also highly active against several human tumour types (Perry *et al.*, 1986; Beer *et al.*, 1987). Carboplatin differs from *cis*-diamminedichloroplatinum(II) (cisplatin) with respect to pharmacological properties such as slower drug elimination due to a different aqueous solubility (Los *et al.*, 1991*a*) and a lower protein binding capacity (McVie *et al.*, 1985), as a result of structural changes of cisplatin (Harland *et al.*, 1984; Zwelling, 1987). The cytotoxicity of platinum compounds is due to reaction of the platinum molecule with nucleophilic sites on the DNA (Meyn *et al.*, 1980; Terheggen *et al.*, 1988). Carboplatin is similar to cisplatin with regard to the type of DNA lesions, which are presumably responsible for the cytotoxicity (Zwelling *et al.*, 1979; Roberts & Friedlos, 1981; Micetich *et al.*, 1985; Knox *et al.*, 1986; Fichtinger-Schepman *et al.*, 1989; Teicher *et al.*, 1991).

Hyperthermic enhancement of effects of cisplatin both *in vitro* (Wallner *et al.*, 1986; Wallner & Li, 1987; Herman *et al.*, 1988) and *in vivo* (Alberts *et al.*, 1980; Overgaard *et al.*, 1991) has generated considerable interest as a therapy for malignancy. Carboplatin also seems to be a promising agent for combination with hyperthermia. Cohen and Robins (1987) found that carboplatin-induced cytotoxicity was enhanced by heat, *in vitro*. Ohno *et al.* (1991) reported that, in rats bearing a fibrosarcoma, the simultaneous combination of carboplatin and whole body hyperthermia produced less toxicity to normal tissues than a similar treatment using cisplatin. The former combination therefore produced an increase in therapeutic gain over that seen with the latter. With respect to timing and sequencing of cisplatin and heat, it is generally accepted that simultaneous drug and heat exposure is most cytotoxic to cultured cells (Wallner *et al.*, 1986; Wallner & Li, 1987). Similarly, the sequence of heat and carboplatin exposure is also one factor which affects the magnitude of the thermal enhancement (Baba *et al.*, 1989). However, there is a paucity in information as to how timing and sequence between heat and carboplatin may influence carboplatin-induced cytotoxicity.

We examined the influence of combining heat and cisplatin or carboplatin exposure on survival of HeLa cells. The effects of heat/drug treatment period and the sequence were examined. We have also studied the relationship between carboplatin-mediated cytotoxicity and intracellular platinum concentrations.

### Materials and methods

### *Drugs*

Cisplatin and carboplatin were obtained from Nippon Kayaku Co. Ltd. (Tokyo, Japan) and Bristol-Myers Squibb Co. (Tokyo, Japan), respectively. The compounds were dissolved just before use in Hanks' balanced salt solution (HBSS), to obtain the designated concentrations.

### *Cells*

The HeLa cells we used were maintained in a monolayer culture in Eagle's minimal essential medium (Nissui Pharmaceutical Co. Ltd., Tokyo, Japan) containing 292 mg ml$^{-1}$ of *L*-glutamine, 100 units ml$^{-1}$ of penicillin, 0.1 mg ml$^{-1}$ of streptomycin and 0.04 mg ml$^{-1}$ of gentamycin supplemented with 10.0% heat inactivated fetal calf serum (GIBCO Laboratories, NY, USA). Cultures were routinely incubated at 37.0°C in a humidified atmosphere of 5.0% $CO_2$ in air.

### *Heat/drug exposure*

Exponentially growing HeLa cells were trypsinised, centrifuged and resuspended in HBSS in a glass test tube (approximately $5 \times 10^5$/0.4 ml HBSS). To investigate the time-dependent response of the cells to treatment, they were exposed to a temperature of 42.8°C for the designated time, before, during or after treatment with 33.2 μM cisplatin or

Correspondence: Y. Maehara, Department of Surgery II, Faculty of Medicine, Kyushu University, 3-1-1 Maidashi, Higashi-ku, Fukuoka 812, Japan.
Received 29 April 1992; and in revised form 9 February 1993.

265 μM carboplatin, for the same period. To investigate heat/drug sequencing, the cells were exposed to heat for 30 min at 42.8°C at varying times before, during or after the start of treatment with either cisplatin or carboplatin at concentrations of 33.2 μM or 265 μM, respectively, for 30 min. These concentrations were equal to the $IC_{50}$s for 30 min treatment with the drugs as determined in dose response survival experiments (Figure 1). The heat exposure involved placing cells in a glass tube and immersing the tube, tightly sealed with a sterile rubber stopper and punctured with needles for inflow and outflow of air, in a water bath heated to a precise temperature. The temperature of the water bath was automatically maintained at 42.8°C within ± 0.1°C. After drug treatments, the cells were washed three times with fresh, drug-free HBSS heated to the same temperature.

### *Survival experiments*

Cell survival assay was carried out using the colony formation method. After heat/drug exposure, the cells were washed three times with HBSS. The number of cells in each group was counted and the cells were plated into 60-mm sterile plastic culture dishes (Corning No. 25010, USA), in triplicate at two dilutions (300 and 500 cells for control; 1,000 and 3,000 cells for each treatment group) and incubated in a humidified atmosphere with 5.0% $CO_2$ at 37.0°C. After 1 week, the colonies were fixed with ethanol, and stained with Giemsa solution. Colonies of 50 or more cells were counted. Each experiment was repeated three times.

### *Determination of intracellular platinum concentrations*

After treatment with 33.2 μM of cisplatin or 265 μM of carboplatin for 30 min, before, during or after 30 min exposure to heat with an interval of 30 min, the cells were placed on ice and washed three times with drug-free HBSS to remove extracellular drug. The cells were then stored in a freezer. The final cell pellet collected was sonicated and the mass of intracellular platinum was determined by atomic absorption spectrophotometry in a pyrocoated graphite cuvette (Hitachi Model 180-7444, Hitachi, Ltd., Tokyo, Japan), using a polarised flameless atomic absorption spectrophotometer (Zeeman Model Z-8000, Hitachi, Ltd., Tokyo, Japan) (Leroy et al., 1977). Platinum atomic absorption standard solution (No. P-6401, Sigma Co., St. Louis, MO, USA) was diluted to two different concentrations and these were run with each experiment for calibration. The concentration of platinum was expressed as μg/$10^7$ cells.

**Figure 1** Single effect of cisplatin (O) or carboplatin (Δ) against HeLa cells for 30 min. Points, means of three independent experiments; bars, s.d.

### *Statistical analysis*

The Welch-Aspin t-test was used to determine significance of the difference in slopes of the time-dependent survival curves (Snedecor & Cochran, 1989; Krag et al., 1990). Comparisons of intracellular platinum concentrations between treatment groups were made using Student's t-test. When the value of P was less than 0.05, the difference was considered to be statistically significant.

### Results

### *Effects of drug alone and drug/heat combination sequencing on HeLa cells*

The value of plating efficiency for control cells was 92.0 ± 7.8%. All the time-dependent survival response curves showed exponential inhibition of the clonogenic activity of HeLa cells. The slopes of time-heat/drug response curves were calculated for cisplatin and carboplatin. The regression coefficients from our data gave slopes of − 0.0430 and − 0.1331 $min^{-1}$ for cisplatin alone, and for simultaneous treatment with cisplatin and heat, respectively. Hence, heat produced a 3.10-fold increase in the slope. The corresponding estimates for carboplatin were − 0.0154 and − 0.0456 $min^{-1}$. Heat therefore gave a 2.96-fold increase in the slope, a value similar to that for cisplatin. The concomitant exposure to heat and cisplatin showed a greater inhibition of the surviving fraction than seen in any of the other groups treated with cisplatin, with significant differences in the slopes ($P < 0.01$), as shown in Figure 2. In case of carboplatin, when exposure to heat was during or prior to carboplatin treatment, the effect on the surviving fraction was greatest, with statistically significant differences in the slopes ($P < 0.01$), as shown in Figure 3.

**Figure 2** Effects of heat alone, cisplatin alone or heat/cisplatin combination on survival of HeLa cells. Symbols show the following treatment: ■, heat (42.8°C) alone; O, 33.2 μM of cisplatin alone; ●, cisplatin followed by heat at 30 min intervals; ▲, heat followed by cisplatin at 30 min intervals; and ◆, simultaneous treatment of cisplatin and heat. The cells were treated with either cisplatin or heat for 30 min. Points, means of triplicate experiments; bars, s.d.

**Figure 3** Effects of heat alone, carboplatin alone or heat/carboplatin combination on survival of HeLa cells. Symbols show the following treatment: ■, heat (42.8°C) alone; Δ, 265 μM of carboplatin alone; ●, carboplatin followed by heat at 30 min intervals; ▲, heat followed by carboplatin at 30 min intervals; and ◆, simultaneous treatment of carboplatin and heat. The cells were treated with either carboplatin or heat for 30 min. Points, means of triplicate experiments; bars, s.d.

**Figure 5** Effects of treatment sequence of heat and carboplatin on survival of HeLa cells. Cells were exposed to a 42.8°C temperaturre for 30 min at varying times before (negative time values), during (time 0), and after (positive time values) a 30 min carboplatin treatment at 265 μM. Points, means of three independent experiments; bars, s.d.

**Figure 4** Effects of treatment sequence of heat and cisplatin on survival of HeLa cells. Cells were exposed to a 42.8°C temperature for 30 min at varying times before (negative time values), during (time 0), and after (positive time values) a 30 min cisplatin treatment at 33.2 μM. Points, means of three independent experiments; bars, s.d.

*Effects of treatment sequence on cell killing by heat and drugs*

Figures 4 and 5 show the effects of drug-heat sequencing and duration on the cytotoxicity of combined treatment with heat and cisplatin or carboplatin, respectively. When the cells were exposed to heat during a 30 min exposure to cisplatin, cell killing was greatest. Cisplatin and heat applied with various time intervals between them had less cytotoxic effect than simultaneous treatment. On the other hand, heat treatment immediately prior to or during exposure of carboplatin was the most cytotoxic. Carboplatin administered prior to heat had less cytotoxic effect.

*Correlation between cytotoxicity and intracellular platinum concentrations in HeLa cells*

Table I shows the intracellular platinum concentration of each combination of heat and drugs, compared with cells treated with the drug alone. For cisplatin, a significantly higher platinum concentration was seen in cells exposed to heat and cisplatin simultaneously, compared with cells treated with heat following or prior to cisplatin ($P < 0.05$). In the case of carboplatin, cells treated with heat before the drug had intracellular concentrations of platinum which were 2.75-fold higher than seen in the drug alone treated group ($P < 0.01$). However, in the case of carboplatin exposure prior to heat treatment, there was a decrease in platinum concentrations to levels lower than seen with the drug alone.

**Table I** The relative values of intracellular concentrations of platinum in HeLa cells exposed to heat before, during and after cisplatin/carboplatin treatment at 30 min intervals

| Treatment | Intracellular platinum concentration[a] Cisplatin[b] | Carboplatin[c] |
|---|---|---|
| Drug + Heat[d] | 1.57 ± 0.11 (1.64[e]) | 2.78 ± 0.14 (1.42) |
| Drug→Heat[d] | 0.88 ± 0.09 (0.92) | 1.31 ± 0.07 (0.67) |
| Heat→Drug[d] | 1.15 ± 0.08 (1.24) | 5.38 ± 0.16 (2.75) |

[a]μg/10[7] cells; [b]33.2 μM; [c]265 μM. [d]The cells were exposed to each drug or to heat for 30 min. [e]Relative value when the intracellular platinum concentration in HeLa cells treated with each drug alone was 1.0.

### Discussion

Heat exposure markedly enhanced the cytotoxicity induced by either cisplatin or carboplatin, findings comparable to results seen in other studies (Micetich *et al.*, 1985; Cohen & Robins, 1987). Our results showed that cisplatin cytotoxicity was most enhanced by heat when these treatments were given simultaneously. Several previous studies have also shown that the simultaneous exposure of cisplatin and heat resulted in the greatest cytotoxic effect (Wallner *et al.*, 1986; Wallner & Li, 1987). On the other hand, in the case of carboplatin, heat exposure immediately prior to or during treatment with carboplatin had the greatest effect on cell survival. Cohen and Robins (1987) have suggested that heat should be given during or immediately prior to carboplatin, in a clinical setting, based on data obtained in laboratory studies.

The mechanisms by which hyperthermia can increase the cytotoxicity of chemotherapeutic agents include increased drug levels in cells (Hahn & Strande, 1976) and hyperthermia induced inhibition of DNA repair (Meyn *et al.*, 1979). Moreover, for antitumour alkylating agents such as cisplatin analogues, a linear relationship was found between DNA cross-link formation and the dose of drugs. The rate of DNA

adduct formation at elevated temperature was higher than seen at normal temperature (Meyn et al., 1980; Los et al., 1982). With regard to the first mechanism mentioned above, other investigators have reported that heat increases the intracellular platinum concentration in cells exposed to cisplatin *in vitro* (Wallner et al., 1986; Los et al., 1991*b*) and *in vivo* (Alberts et al., 1980) and our data show that the heat-induced increase in intracellular platinum concentrations correlates with decrease in the survival of HeLa cells.

However, the decrease in survival of HeLa cells which we have observed cannot be explained by increased platinum concentrations alone. Firstly, although heat exposure prior to and during carboplatin was equally toxic (Figure 3), the uptake of carboplatin into cells was higher after heat prior to carboplatin treatment than after simultaneous heat and carboplatin treatment. Simultaneous heat and carboplatin produced a 2.96-fold greater effect on cell survival but only a 1.42-fold increase in intracellular platinum levels (Table I). On the other hand, the increased effects seen for heat prior to carboplatin were almost identical (2.85 *vs* 2.75).

Secondly, although the drug given alone and immediately prior to heat exposure were almost equally cytotoxic (Figures 2 and 3), platinum levels in cells treated with heat prior to drug exposure were lower than for drug alone (Table I). The first point may mean that the carboplatin-induced cytotoxicity could be a result of an increased rate of DNA lesion formation during heating. Another possible explanation is that heat increases cell membrane permeability to drugs (Arancia et al., 1989) and the membrane transport of drugs (Hahn et al., 1975) thereby altering cellular metabolism (Hahn & Shiu, 1983). Based on these reasons, the overall kinetics of carboplatin may change. The second observation above could possibly be explained if heat increases drug excretion before formation of DNA lesions. Micetich et al. (1985) have shown that DNA adduct formation reaches a maximum several hours after cisplatin or carboplatin exposure. Although the time course for carboplatin is thought to be different from that for cisplatin (Micetich et al., 1985; Knox et al., 1986; Los et al., 19991*b*), the relatively short intervals which we used in our study did not show any difference in effects between carboplatin before heat treatment and cisplatin before heat treatment.

Various side effects result from combined use of heat and anticancer drugs in a clinical setting, including increased nephrotoxicity by cisplatin (Gonzales-Vitale et al., 1977; Campbell et al., 1983) and myelosuppression by carboplatin (Curt et al., 1983; Sternberg et al., 1985). However, optimal use of heat/drug scheduling shows promise for combination treatment of cancer patients, as reported by Baba et al. (1989). Our findings may be helpful in the design of combined treatments with hyperthermia and cisplatin analogues.

This research was supported in part by Grants-in-Aid for cancer research from the Ministry of Health and Welfare, Japan (2-18) and from the Fukuoka Cancer Society.

We thank M. Ohara for critical comments and B.A. Teicher, Ph.D., Dana-Farber Cancer Institute, for helpful discussions.

### References

ALBERTS, D.S., PENG, Y.M., CHEN, G., MOON, T.E., CETAS, T.C. & HOESCHELE, J.D. (1980). Therapeutic synergism of hyperthermia-*cis*-platinum in a mouse tumor model. *J. Natl Cancer Inst.*, **65**, 455-461.

ARANCIA, G., CRATERI TROVALUSCI, P., MARIUTTI, G. & MONDOVI, B. (1989). Ultrastructural changes induced by hyperthermia in Chinese hamster V79 fibroblasts. *Int. J. Hyperthermia*, **5**, 341-350.

BABA, H., SIDDIK, Z.H., STREBEL, F.R., JENKINS, G.N. & BULL, J.M.C. (1989). Increased therapeutic gain of combined *cis*-diamminedichloroplatinum(II) and whole body hyperthermia therapy by optimal heat/drug scheduling. *Cancer Res.*, **49**, 7041-7044.

BEER, M., CAVALLI, F., KAYE, S.B., LEV, L.M., CLAVEL, M., SMYTH, J., GLABBEKE, M.V., RENARD, J. & PINEDO, H.M. (1987). A phase II study of carboplatin in advanced or metastatic stomach cancer. *Eur. J. Cancer Clin. Oncol.*, **23**, 1565-1567.

CAMPBELL, A.B., KALMAN, S.M. & JACOBS, C. (1983). Plasma platinum levels: relationship to cisplatin dose and nephrotoxicity. *Cancer Treat. Rep.*, **67**, 169-172.

COHEN, J.D. & ROBINS, H.I. (1987). Hyperthermic enhancement of *cis*-diammine-1,1-cyclobutane dicarboxylate platinum(II) cytotoxicity in human leukemia *in vitro*. *Cancer Res.*, **47**, 4335-4337.

CURT, G.A., GRYGIEL, J.J., CORDEN, B.J., OZOLS, R.F., WEISS, R.B., TELL, D.T., MYERS, C.E. & COLLINS, J.M. (1983). A phase I and pharmacokinetic study of diamminecyclobutanedicarboxylate platinum (NSC 241240). *Cancer Res.*, **43**, 4470-4473.

FICHTINGER-SCHEPMAN, A.M.J., VENDRICK, C.P.J., VAN DIJK-KNIJINENBURG, W.C.M., DE JONG, W.H., VAN DER MINNEN, A.C.E., CLAESSEN, A.M.E., VAN DER VELDE-VISSER, S.D., DE GROOT, G., WUBS, K.L., STEERENBERG, P.A., SCORNAGEL, J.H. & BERENDS, F. (1989). Platinum concentrations and DNA adduct levels in tumors and organs of cisplatin-treated LOU/M rats inoculated with cisplatin-sensitive or -resistant immunoglobulin M immunocytoma. *Cancer Res.*, **49**, 2862-2867.

GONZALES-VITALE, J.C., HAYES, D.M., CVLTKAVIC, E. & STERNBERG, S.S. (1977). The renal pathology of *cis*-platinum-II. *Cancer*, **39**, 1372-1381.

HAHN, G.M., BRAUN, J. & HAR-KEDAR, I. (1975). Thermochemotherapy: synergism between hyperthermia (42-43°C) and adriamycin (or bleomycin) in mammalian cell inactivation. *Proc. Natl Acad. Sci. USA*, **172**, 937-940.

HAHN, G.M. & STRANDE, D.P. (1976). Cytotoxic effects of hyperthermia and adriamycin on Chinese hamster cells. *J. Natl Cancer Inst.*, **37**, 1063-1067.

HAHN, G.M. & SHIU, E.C. (1983). Effect of pH and elevated temperatures on the cytotoxicity of some therapeutic agents on Chinese hamster cells *in vitro*. *Cancer Res.*, **43**, 5789-5791.

HARLAND, S.J., NEWELL, D.R., SIDDIK, Z.H., CHADWICK, R., CALVERT, A.H. & HARRAP, K.R. (1984). Pharmacokinetics of *cis*-diammine-1,1-cyclobutane dicarboxylate platinum(II) in patients with normal and impaired renal function. *Cancer Res.*, **44**, 1693-1697.

HARRAP, K.R., JONES, M. & WILKINSON, C.R. (1980). Antitumor, toxic and biochemical properties of cisplatin and eight other platinum complexes. In *Cisplatin: Current Status and New Developments*. Prestayko, A.W., Crooke, S.T. & Carter, S.K. (eds) pp. 193-212, Academic Press: New York.

HERMAN, T.S., TEICHER, B.A., CATHCART, K.N.S., KAUFMANN, M.E., LEE, J.B. & LEE, M.N. (1988). Effect of hyperthermia on *cis*-diamminedichloroplatinum(II) (Rhodamine 123) $_2$[tetrochloroplatinum(II)] in a human squamous cell carcinoma line and a *cis*-diamminedichloroplatinum(II)-resistant subline. *Cancer Res.*, **48**, 5101-5105.

KNOX, R.J., FRIEDLOS, F., LYDALL, D.A. & ROBERTS, J.J. (1986). Mechanism of cytotoxicity of anticancer platinum drugs: evidence that *cis*-diamminedichloroplatinum(II) and *cis*-diammine-(1,1-cyclobutanedicarboxylato)platinum(II) differ only in the kinetics of their interaction with DNA. *Cancer Res.*, **46**, 1972-1979.

KRAG, D.W., THEON, A.P. & GAN, L. (1990). Hyperthermic enhancement of Rhodamine 123 cycotoxicity in B16 melanoma cells *in vitro*. *Cancer Res.*, **50**, 2385-2389.

LEROY, A.F., WEHLING, M.L., SPONSELLER, H.L., FRIAUF, W.S., SOLOMON, R.E. & DEDRICK, R.L. (1977). Analysis of platinum in biological materials by flameless atomic absorption spectrophotometry. *Biochem. Med.*, **18**, 184-191.

LOS, G., SMINIA, P., WONDERGEM, J., MUTASAERS, P.H.A., HAVEMEN, J., TEN BOKKEL HUININK, D., SMALS, O.A.G., GONZALEZ-GONZALEZ, D. & MCVIE, J.G. (1991*a*). Optimization of intraperitoneal cisplatin therapy with regional hyperthermia in rats. *Eur. J. Cancer*, **27**, 472-477.

LOS, G., VERDEGAAL, E., NOTEBORN, H.P.J.M., RUEVENKAMP, M., DE GRAAF, A., MEESTERS, E.W., TEN BOKKEL HUININK, D. & MCVIE, J.G. (1991*b*). Cellular pharmacokinetics of carboplatin and cisplatin in relation to their cytotoxic action. *Biochem. Pharmacol.*, **42**, 357-363.

LOS, G., SMALS, O.A.G., VAN VUGT, M.J.H., VAN DER VLIST, M., DEN ENGELSE, L., MCVIE, J.G. & PINEDO, H.M. (1992). A rationale for carboplatin treatment and abdominal hyperthermia in cancers restricted to the peritoneal cavity. *Cancer Res.*, **52**, 1252-1258.

McVie, J.G., ten Bokkel Huinink, W., Dubbelman, R., Franklin, H., van den Vijgh, W. & Klein, I. (1985). Phase I study and pharmacokinetics or intraperitoneal carboplatin. *Cancer Treat. Rev.*, **12**, 35-41.

Meyn, R.E., Corry, P.M., Fletcher, S.E. & Demetriades, M. (1979). Thermal enhancement of DNA strand breakage in mammalian cells treated with bleomycin in rats. *Int. J. Radiat. Oncol. Biol. Phys.*, **5**, 1487-1489.

Meyn, R.E., Corry, P.M., Fletcher, S.E. & Demetriades, M. (1980). Thermal enhancement of DNA damage in mammalian cells treated with *cis*-diamminedichloroplatinum(II). *Cancer Res.*, **40**, 1136-1139.

Micetich, K.C., Barnes, D. & Erickson, L.C. (1985). A comparative study of the cytotoxicity and DNA-damaging effects of *cis*-(diammine)(1,1-cyclobutanedicarboxylato)-platinum(II) and *cis*-diamminedichloroplatinum(II) on L1210 cells. *Cancer Res.*, **45**, 4043-4047.

Ohno, S., Siddik, Z.H., Baba, H., Stephens, L.C., Strebel, F.R., Wondergem, J., Khokhar, A.R. & Bull, J.M.C. (1991). Effect of carboplatin combined with whole body hyperthermia on normal tissue and tumor in rats. *Cancer Res.*, **51**, 2994-3000.

Overgaard, J., Radacic, M.M. & Gran, C. (1991). Interaction of hyperthermia and *cis*-diamminedichloroplatinum(II) alone or combined with radiation in a C3H mammary carcinoma *in vivo*. *Cancer Res.*, **51**, 707-711.

Perry, D.J., Weiss, R.B., Creekmore, S.P., Micetich, K.C. & Curt, G.A. (1986). Carboplatin for advanced colorectal carcinoma: a phase II study. *Cancer Treat. Rep.*, **70**, 301-302.

Roberts, J.J. & Friedlos, F. (1981). Quantitative aspects of the formation and loss of interstrand cross-links in Chinese hamster cells following treatment with *cis*-diamminedichloroplatinum(II) (cisplatin). *Biochem. Biophys. Acta*, **655**, 146-151.

Snedecor, G.W. & Cochran, W.G. (1989). The comparison of two samples. In *Statistical Methods, Ed. 7*, Ames, I.A. (ed) pp. 83-106, Iowa State University Press.

Sternberg, C., Kelsen, D., Dukeman, M., Leichman, L. & Heelan, R. (1985). Carboplatin: a new platinum analog in the treatment of epidermoid carcinoma of the esophagus. *Cancer Treat. Rep.*, **69**, 1305-1307.

Teicher, B.A., Pfeffer, M.R., Alvarez Sotomayor, E. & Herman, T.S. (1991). Schedule dependent tumour growth delay, DNA cross-linking and pharmacokinetic parameters in target tissues with *cis*-diamminedichloroplatinum(II) and etanidazole with or without hyperthermia or radiation. *Int. J. Hyperthermia*, **7**, 773-784.

Terheggen, P.M.A.B., Dijkman, R., Begg, A.C., Dubbelman, R., Floot, B.G.J., Hart, A.A.M. & den Engelse, L. (1988). Monitoring of interaction products of *cis*-diamminedichloroplatinum(II) and *cis*-diammine(1,1-cyclobutane-dicarboxylato) platinum(II) with DNA in cells from platinum-treated patients. *Cancer Res.*, **48**, 5597-5603.

Wallner, K.E., DeGregoria, M.W. & Li, G.C. (1986). Hyperthermic potentiation of *cis*-diamminedichloroplatinum(II) cytotoxicity in Chinese hamster ovary cells resistant to the drug. *Cancer Res.*, **46**, 6242-6245.

Wallner, K.E. & Li, G.C. (1987). Effect of drug exposure duration and sequencing on hyperthermic potentiation of mitomycin-C and cisplatin. *Cancer Res.*, **47**, 193-495.

Zwelling, L.A., Anderson, T. & Kohn, K.W. (1979). DNA-protein and DNA interstrand cross-linking by *cis*- and *trans*-platinum(II) diammine-dichloride in L1210 mouse leukemia cells and relation to cytotoxicity. *Cancer Res.*, **39**, 365-369.

Zwelling, L.A. (1987). Cisplatin and new platinum analogs. In *Cancer Chemotherapy and Biological Response Modifiers Annual 9*, Pinedo, H.M., Longo, D.L. & Chabner, B.A. (eds) pp. 71-78. Elsevier Science Publishers, B.V.: Amsterdam.